# Clinical Lecture in the U.S. Marine Hospital

**Published:** 1852-12

**Authors:** W. B. Herrick


*Clinical Lecture, in the U. S. Marine Hospital, by* W. B. Herrick, M. D.

In a previous lecture we gave our definition of assimilation and excretion, and also pointed out their separate stages. We propose now to examine the physiological pathology of each of these processes, commencing with primary assimilation, and as we proceed shall use cases in the wards to illustrate our views of meeting the several indications.

We may, for the sake of convenience, divide this process into two stages, viz., mastication and solution of the food, and its absorption into the lacteals and veins. It is evident that there may be morbid phenomena arising from imperfect performance of either of these functions. There are certain conditions in which it is impossible for solid food to be perfectly prepared for introduction into the stomach, as in mechanical defect of the dental organs, or the muscles concerned in mastication. There may also be disease of the fauces or æsophagus, and paralysis of the muscles of deglutition. In such cases the indications are of course to introduce nutriment in a fluid state, in the natural manner if possible; but, if necessary, through a stomach tube. If this is not feasible, we may introduce it per rectum in such a condition that it may be absorbed from the lower intestines. For this purpose we should select saccharine, starchy, or albuminous substances, for reasons which we shall see hereafter.

The saliva secreted during mastication is of two kinds, and seems intended to subserve different purposes. That from the sublingual and parotid glands has the consistence and appearance of water, and seems to be designed principally to saturate the food, and thus to favor its mechanical division; while that from the submaxillary glands is thick and viscid, and seems mainly to facilitate deglutition. The mixed saliva also possesses the property of acting to some extent on amylaceous substances, tending to change them into sugar. M. Bernard has concluded, that the agent producing these changes in starch is not different from some other nitrogenized matter undergoing change. This property of the saliva is destroyed by acids. You will see from what we have said that the condition of the bolus, introduced into the stomach, may be modified very much by the manner in which these glands perform their functions. An arrest of the salivary secretion would render necessary for the solution of the food an increased amount of the gastric secretion. If, then, there be a deficiency of saliva, our object will be to excite the glands by the administration of substances which are known to have an influence over them; such, for instance, as the mineral acids which seem to act chemically by their affinity for the alkaline secretion, or by the use of salines which probably favor exosmosis, and generally by the administration of substances containing Iodine or Chlorine, agents known to possess a specific influence over secreting structures. You have all observed in the Hospital instances in which a dry tongue and mouth has been moistened, and all the secretions rendered abundant in a very short time under the influence of Chloride of Sodium, or Nitro. Mur. Acid; the same results may be accomplished by the use of the Chlorides or Iodides of Mercury, Potassium, and, perhaps, other Haloid compounds.

The food having been received into the stomach may have morbid phenomena from two distinct sets of causes: First, there may be want of muscular action of the walls of the organ, from which it happens that the alimentary mass is not brought in contact properly with the secreting surfaces, and intimately grinded with the gastric juice. The process of solution is therefore slow, and more or less imperfect. This deficient action may not be confined to the stomach, but extend through the whole length of the alimentary tube, giving rise to constipation primarily, and secondarily to all those accidents dependent upon it. In such cases we may direct our efforts either to the muscular structure of the organs, by giving those medicines that excite muscular contraction, such for instance as strychnia and general tonics; or we may act immediately on the food by administering solvents for it, as the Muriatic and other acids which are known to possess the power of dissolving albumen. The Nitro. Mur. Acid, as you have often seen it prescribed here, generally has the effect to keep the bowels open, and we are sometimes obliged to stop its use, from the fact of its producing too free evacuations. Another class of morbid phenomena depend on an excess or deficiency of the secretions proper to the tube, and those coming into it from glandular organs, as from the liver and pancreas. The capacity of the stomach to digest food will depend evidently on the quantity and quality of the gastric juice, as well as upon the muscular activity of the organ. In the affection commonly known as dyspepsia, difficult digestion, we may have one or both of these conditions, and our indications of treatment will be modified accordingly. If there is already circulating in the blood-vessels a sufficiency of nutriment we may reduce the quantity of food to the capacity of the stomach, while at the same time we endeavor to restore to the organ its functions by the administration of tonics and stimulants if there be debility, and of substances exciting the secretions if they are defective; and, on the other hand, we administer a quality of food for the introduction of which into the system the action of the stomach is not required.

In order that we may be understood we may here remark, that there are three classes of substances entering into the composition of the tissues and fluids of the body, viz.: albuminous, carbonaceous or hydro-carbonaceous, and mineral, consisting mainly of Lime, Potash, Soda, Magnesia, and Iron. Albumen, salts, and the starchy portions of the carbonaceous elements are introduced through the mesenteric veins and, with the exception of the minerals, require the action of the juices of the stomach; while oils, which subserve the same purpose in the economy as starch, pass through the stomach unchanged, till, meeting with the pancreatic fluid, an alkaline secretion, they are formed into an emulsion which is taken up and introduced into the blood-vessels through the lacteals. The final disposition of these classes of nutriment is also various. The carbonaceous substances, both fatty and amylaceous, serve as respiratory food, and also furnish the fat which is deposited in the cellular tissues. Albumen and the minerals, on the other hand, enter into the composition of the solids, and are designed mainly for the manufacture, so to speak, of the different structures.

You will see, therefore, why we have prescribed oils in cases where there was faulty action of stomach, or obstruction of the portal circle, with, at the same time, no evidences of deficiency of the pancreatic secretion; on the contrary, albumen, saccharine, and starchy substances are indicated when from any cause the pancreas does not perform its functions.

The biliary matter modifies no doubt, to a certain extent, the digestive process, especially below the opening of the hepatic duct. A deficiency of this material may probably, from the want of the stimulus which it affords, result in constipation; if, however, this deficiency is dependent on the retention of the bile in the ducts, rather than upon defective action of the secreting cells, we are more likely to have congestion of the capillaries of the vena portarum, with consequent engorgement of the larger mesenteric veins, and finally copious watery discharges; or we may have, as in the first instance, constipation from the want of the stimulus which the bile furnishes, primarily, with diarrhœa from portal congestion, secondarily. This is a vice of primary assimilation, which you will not unfrequently be called upon to treat. It is evident that the therapeutical indications are to maintain in the terminal branches of the biliary ducts a soluble condition of their proper secretion, consisting mainly of fatty acids. This is accomplished normally by the union of an alkali, soda supplied by the arterial branches. This fact directs us to the means of fulfilling the indications, and it has been found that alkalies and especially soda, are efficient in correcting the morbid conditions to which we have alluded.

The small glands of the intestines are subject to disease, but whether they affect nonprimary assimilation or secondary secretion is a question which, in the present state of our knowledge, is undetermined.

We have alluded to the office of the lacteals as the bearers of fatty materials alone; they do, however, absorb from the intestines some other substances, but which are secreted from the chyle, according to M. Bernard, by the mesenteric glands, and probably transferred by them to the mesenteric veins. It seems, therefore, to be the office of these glands, in part, to prevent from passing into the blood any substances which are imperfectly prepared, and which require for their elaboration the action of the liver. Hence it is, that in diseases of these glands the purest oils, which on their part require the least action, are indicated.

